# TAPAS—A Prospective, Multicentre, Long-Term Cohort Study in Children, Adolescents and Adults with Seasonal Allergic Rhinitis—Design and Early Results

**DOI:** 10.3390/jcm14082609

**Published:** 2025-04-10

**Authors:** Michael Gerstlauer, Julia Hiller, Jennifer Raab, Katrin Birkholz, Martin Tapparo, Christian Neuhof, Laura Day, Anna Rybachuk, Cengizhan Acikel, Hacer Sahin, Kim Hebbeler, Sven Becker, Christian Vogelberg, Silke Allekotte, Matthias F. Kramer, the TAPAS Study Group

**Affiliations:** 1 Paediatric and Adolescent Medicine, Department of Paediatric Pneumology and Allergology, University Medical Center Augsburg, 86156 Augsburg, Germany; 2 Bencard Allergie GmbH, 80804 Munich, Germany; 3 ClinCompetence Cologne GmbH, 50668 Cologne, Germany; 4 Institute of Medical Statistics and Computational Biology, University of Cologne, 50923 Cologne, Germany; 5 Department of Otorhinolaryngology, Head and Neck Surgery, University of Tübingen, 72074 Tübingen, Germany; 6 Department of Pediatric Pneumology and Allergology, Faculty of Medicine, University Hospital Carl Gustav Carus Dresden, Technische Universität Dresden, 01069 Dresden, Germany; 7 Allergy Therapeutics, Worthing BN14 8SA, UK

**Keywords:** allergen-specific immunotherapy, subcutaneous immunotherapy, allergic rhinitis, asthma, paediatric allergy, microcrystalline tyrosine-adsorbed allergoids, cohort study, disease-modifying effect

## Abstract

**Background/Objectives**: The guideline on allergen-specific immunotherapy of the European Academy of Allergy and Clinical Immunology recommends subcutaneous allergen-specific immunotherapy for the treatment of allergic rhinitis in children and adults with moderate to severe symptoms. The five years cohort study described below was designed in 2020 to demonstrate non-inferiority in terms of safety, tolerability and efficacy in a paediatric population compared with adult patients treated with microcrystalline tyrosine-adsorbed allergoids for their tree and grass pollen allergy in a perennial setting. Here, we present the preliminary findings from the first year. **Methods**: The Combined Symptom and Medication Score was chosen as the primary endpoint of this therapy. Secondary endpoints include the Rhinoconjunctivitis Quality of Life Questionnaire, the retrospective Rhinoconjunctivitis score, the Asthma Control Test and the Rhinitis Control Test, as well as an analysis of adverse drug reactions. **Results**: A total number of 320 patients were enrolled into this study, with 129 of these patients in the age group between 5 and 17 years and 191 patients in the adult age group. Mean Combined Symptom and Medication Score values did not differ significantly between minors and adults in the first pollen season after treatment induction. The retrospective score showed a strong and significant reduction in rhinoconjunctivitis and asthma symptoms. Treatment was well tolerated, with more than 80% of patients reporting no adverse drug reactions. **Conclusions**: The validity of this study approach of a cohort study has been confirmed by this first interim analysis for the initial course of therapy in the first year.

## 1. Introduction

Allergen-specific immunotherapy (AIT) remains underutilised in the paediatric population. Nevertheless, paediatric patients benefit most from the therapy, not only by reducing symptoms but also by preventing the allergic march from monosensitisation to poly-allergies and from rhinitis to asthma.

The guideline on allergen-specific immunotherapy (AIT) of the European Academy of Allergy and Clinical Immunology (EAACI), based on a meta-analysis conducted by Dhami [1], recommends subcutaneous allergen-specific immunotherapy (SCIT) for the treatment of allergic rhinitis (AR) in children and adults with moderate to severe symptoms that are only suboptimally controlled despite pharmacotherapy [2]. The recommendation grade for both pre-seasonal and perennial SCIT for seasonal AR is slightly higher for adults (grade A) than for children (grade B), as the evidence for children is based exclusively on an open, but randomised, controlled trial (RCT) [3].

Many SCIT studies in which children were also examined are older and were not included in this EAACI analysis due to changes in the quality requirements for studies. In addition, only a few children were included in the studies, or no specific paediatric analyses of the studies were published.

Most clinical trials evaluating the efficacy of AIT only observe the study participants for one or two years under therapy [4], although the European Medicines Agency (EMA) currently recommends an experimental, randomised, controlled design with a duration of three years of therapy with a follow-up period of two years without treatment. Such studies are intended to demonstrate the sustained efficacy and disease-modifying effects of AIT. The active AIT treatment of children, especially with SCIT products, in the context of double-blind, placebo-controlled studies over three years and a subsequent follow-up over two years to determine the long-term effectiveness of the treatment, is not feasible from the point of view of many investigators due to a lack of acceptance by the parents concerned [5]. In such a study setting, some of the children would be treated with a placebo for three years and receive no treatment for a further two years, which could lead to the progression of the disease. Innovative study designs have been proposed to overcome this dilemma [6,7], and a first SCIT trial, meeting the requirements of an EMA Paediatric Investigation Plan (PIP), is currently conducted throughout central Europe (EUCT 2023-508520-36-00).

In daily practice children, adolescents and adults are treated with identical products—in identical dosage and posology. The reason behind this is the general assumption that the immune system is mature by the age of five. Although there is no reason to doubt this assumption, data are desired to support this hypothesis.

This evidence base has improved significantly in the last two decades for sublingual allergen-specific immunotherapy (SLIT) against grass pollen allergy for paediatric populations [8] but remains poor for other allergens [9]. Another problem is that there are decreasing numbers of products marketed in central Europe, especially for children. Glutaraldehyde-modified and microcrystalline tyrosine-adsorbed allergoids (MATAs) are among the few therapeutic allergens authorised for subcutaneous application from age five onwards. MATAs are characterised by the fact that they combine a combination of modified allergens, so-called allergoids, with the biodegradable depot adjuvant Microcrystalline Tyrosine (MCT). This is intended both to improve tolerability compared to native allergens and to increase efficacy through the adjuvant. MATAs have been introduced in numerous countries for many decades and are authorised in Germany, for example, from the age of five for the treatment of allergic rhinitis induced by pollen [10]. The preparations were authorised in Germany as early as 1976 (TA top Gräser) and 1995 (TA top Bäume).

A recent meta-analysis [11] summarised the more than 40 studies that have been conducted with these preparations since the 1960s. Some double-blind, placebo-controlled, randomised prospective studies were also identified. Consequently, the evidence basis for the MATA brand can be described as favourable. The meta-analysis was able to show convincing efficacy data and a superior level of safety and tolerability. Additionally, real-world evidence, based on prescription data, recently demonstrated the long-term benefits of this therapeutic approach, including a reduction in the onset of newly diagnosed asthma, asthma and rhinitis medication [12]. However, data in the paediatric and adolescent age groups were relatively scarce. To address the above-mentioned gaps in knowledge, a controlled non-inferiority approach was chosen to compare children and adolescents with adults regarding allergy-related health outcomes. To this end, the cohort study described below was designed in 2020 to demonstrate non-inferiority in terms of safety, tolerability and efficacy in a paediatric population compared with adult patients.

## 2. Materials and Methods

### 2.1. The Study Design

The aim of this study is to show that the long-term effect of SCIT treatment using the MATA platform (Allergoid TA, Bencard Allergie GmbH, Munich, Germany) has a comparable effect in paediatric and adult patients.

This study was designed prospectively and intended to include around 320 patients, equally divided between the age groups of children/adolescents and adults. Following the principle of non-intervention, usual allergological medical care was a prerequisite. Therefore, only patients suffering from allergic rhinoconjunctivitis with or without mild to moderate bronchial asthma caused by grass pollen or tree pollen and for whom the decision to carry out year-round AIT with the products TA top Gräser or TA top Bäume in Germany had already been made could participate in this non-interventional study (NIS). As a consequence of this NIS setting, prospective data generation could only address the time span from treatment initiation onwards, whereas comparison against baseline could only be realised by retrospective approaches. The usual three-year treatment course is followed by a two-year observation phase without further AIT therapy, aiming to evaluate disease-modifying effects of the MATA products. This phase of the long-term cohort study is still ongoing. The basic design of this NIS therefore corresponds to an EMA PIP, with the difference being using children as controls for adults. The advantage of this approach is that all patients included in this cohort study received effective AIT treatment.

**Figure 1:**
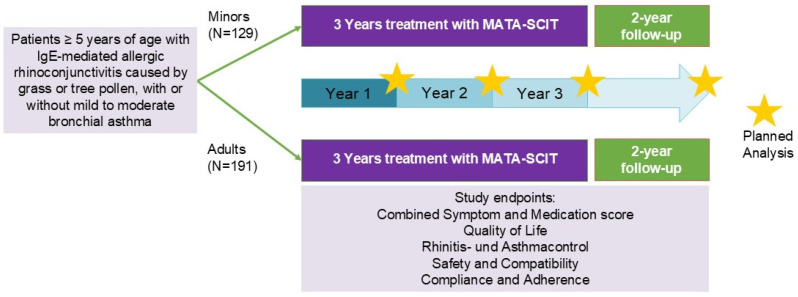
Study design: a total of 320 minors and adults were recruited in 31 study centres in Germany. SCIT: subcutaneous immunotherapy.

The overall design of the trial is depicted in Figure 1. Here, we present the results after the first year of subcutaneous AIT.

### 2.2. Endpoints

The Combined Symptom and Medication Score (CSMS) recommended by the EAACI [13] was chosen as the primary endpoint of this therapy. Data were collected over a one-month observation phase during the respective pollen seasons using a smartphone app V2.023. In the paediatric population a slightly modified score was used to account for the contraindication of systemic glucocorticosteroid treatment as described in [14].

Other secondary objectives of this study included the assessment of quality of life using the Rhinoconjunctivitis Quality of Life Questionnaire (RQLQ(s), AdoIRQLQ, PRQLQ) [15,16,17], pre-seasonally and at the peak of the respective pollen season, as well as asthma symptoms and the Asthma Control Test (ACT) [18,19] in asthmatic patients. The Rhinitis Control Test (RCT) [20,21] was also recorded. Symptoms of allergic rhinoconjunctivitis were retrospectively assessed before the start of each pre-seasonal AIT cycle using a well-established scale [22]. In terms of safety and tolerability, local and systemic reactions that occurred during the treatment interval were recorded, and late symptoms reported by the patients were documented.

This study included the following visits:

- Study inclusion at visit 1
- During active AIT treatment (years 1 to 3), 3 visits were made in year 1 for basic treatment and at least 3 visits per year for continued treatment (on average, 10 injections per year). In years 2 and 3, the maintenance dose is continued at intervals of 4–6 weeks. In general, all injections but also unscheduled visits were documented.
- During the follow-up period in years 4 and 5, 2 visits are planned each year (before the start of the respective pollen season and at the peak of the respective pollen season).

Table S1 in the Supplemental Material provides an overview of the design of this study with the main assessment parameters recorded and the times at which they were collected.

### 2.3. The Patients

At the physician’s discretion, male and female patients were offered participation in this study. Inclusion and exclusion criteria, such as age five years and older and allergic rhinitis, conjunctivitis and/or mild to moderate asthma caused by an IgE-mediated allergy to grass or birch, alder and hazel pollen, strictly met the Summary of Product Characteristics.

### 2.4. Pollen Data

A 14-day peak pollen period in April for tree allergy sufferers and in June for grass allergy sufferers was identified on the basis of daily forecast data from the German weather service DWD. For this period, the non-inferiority of the treatment results in minors compared to adults was to be determined regarding symptom burden and consumption of rescue medication utilising the CSMS.

### 2.5. Statistical Methods

#### 2.5.1. Sample Size Estimation

Non-inferiority is considered to be given for a difference between the daily CSMS of the adults averaged over one month and that of the children/adolescents, which is at most 40% of the averaged standard deviation of the parameter in favour of the adults. The non-inferiority margin was defined by adhering to the guidelines established by the European Medicines Agency (EMA) [23] and the U.S. Food and Drug Administration (FDA) [24]. This margin was determined with the objective of identifying a threshold that reflects a clinically insignificant difference. Due to the limited clinical research directly comparing outcomes between adolescents and adults in this area, expert opinion was sought to guide this determination. For a one-sided test (child values better or at most 40% of the standard deviation worse than adult values) at the 95% level with a power of 90%, 108 analysable patients per group are required after three years, i.e., a total of 216 patients.

Assuming an adherence rate of 70% over 3 years in this study situation, 308 patients are sufficient to be included in the two cohorts. The 12 additional patients serve to compensate for the lack of data in the electronic diary (around 4%, which experience has shown to be sufficient).

Therefore, 320 patients are to be included in this observational study, divided equally into the two cohorts for children/adolescents and adults.

#### 2.5.2. Evaluation Times

After each year of treatment, interim evaluations of the primary target parameter will be carried out to analyse sustained efficacy during long-term treatment, respectively, disease-modification or carry-over effects in the follow-up period.

#### 2.5.3. Primary Endpoint

The effectiveness of the yearly treatment course using TA Gräser top or TA Bäume top is assessed on the basis of the symptom severity and the medication consumption documented by the patient according to the CSMS measured at the peak of the respective pollen season.

The CSMS+ Diary App queries the symptom severity of the individual allergy symptoms sneezing, runny nose, itchy nose, blocked nose, watery eyes, and itchy eyes to calculate the rhinoconjunctivitis daily symptom score (dSS) and the medication intake for daily medication score calculation (dMS). Data collection via the CSMS+ Diary App is described in the Supplemental Material. For the statistical analysis, the primary endpoint, the CSMS, was analysed exploratively. All other parameters were analysed descriptively.

Continuous variables are presented using descriptive statistics (number, mean, standard deviation, median, minimum, maximum, interquartile range and missing values). Categorical data are visualised using absolute and percentage frequencies. No formal statistical tests will be performed except to examine the non-inferiority of the paediatric population on the primary outcome parameter, but two-sided 95% confidence intervals will be used to quantify the accuracy of the results. Subcollectives can be analysed exploratively. The results of both cohorts for the first year of treatment are reported here.

### 2.6. Ethical and Regulatory Framework

This study was carried out in accordance with the German Medicinal Products Act (Arzneimittelgesetz, AMG), Section 67, Subsection 6. After counselling on professional regulations, it was approved by the competent ethics committee of the Cologne University, Cologne, Germany, and registered under the number 20-1417_1-NIS. This study was further registered with ClinicalTrials.gov under the registry number NCT05186025. This study protocol was submitted in 2020 to the competent regulatory agency Paul-Ehrlich-Institut under the number 561. All patients and/or parents provided written informed consent before study inclusion.

## 3. Results

Recruiting paediatric individuals into clinical studies is a challenging task compared to adults. In the first recruitment period in 2020, it was not possible to include the full number of patients in this study. The decision was, therefore, made to continue recruitment in the following pre-seasonal period. This enabled the enrolment of the full planned number of 320 patients into this study, with 129 of these patients in the age group between 5 and 17 years and 191 patients in the adult age group, i.e., 18 years and older. Here, we present an interim analysis of the data of patients in both cohorts after one course of pre-seasonal treatment. Table 1 presents comprehensive baseline characteristics of the patient population.

Demographic data and the distribution between the two cohorts are shown in Table 2.

There were only a few dropouts in the first year, far fewer than known from other real-world studies [25]. The number and reasons for dropping out of this study are shown in the Supplemental Material Tables S2 and S3.

Data on RCAT and ACT will be reported in upcoming publications on the complete study period of five years.

**Figure 2:**
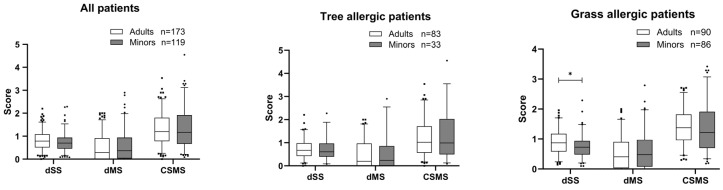
Daily Symptom Score (dSS), Daily Medication Score (dMS) and Combined Symptom and Medication Score (CSMS) during the first pollen season in 2021 or 2022 in the adults and minors group. Data are presented as a box-whisker plot with 5 and 95 percentiles for all patients, for tree pollen allergic patients and for grass pollen allergic patients. * *p* < 0.05 in comparison to adults.

The median CSMS score for all patients after one year was 1.18, and it was slightly higher for adult patients (1.19) than for paediatric patients (1.17). No differences between adults and minors were observed for the dSS and dMS. The picture is similar for grass and tree pollen-allergic patients, for adults and minors, respectively. The visualisation of the primary target parameter can be found in Figure 2.

**Figure 3:**
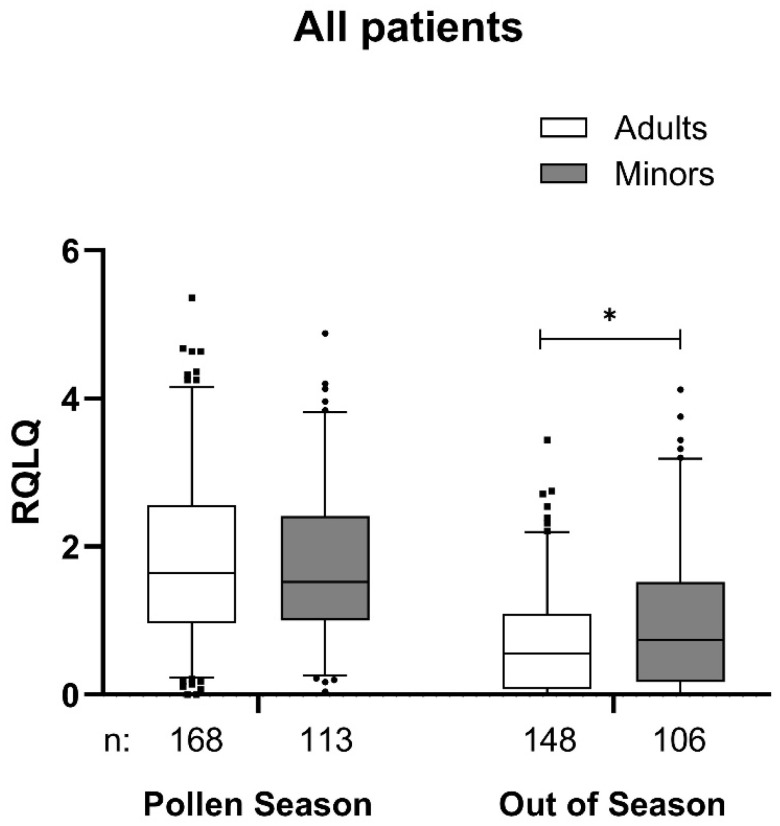
RQLQ Score at the first pollen season in 2021 or 2022 and out of season in the adults and minors group. Data are presented as a box-whisker plot with 5 and 95 percentiles. * *p* < 0.05 in comparison to adults. RQLQ, rhinoconjunctivitis quality of life questionnaire.

The data from the RQLQ health-related quality of life survey in the first year of treatment demonstrate comparable values for both age groups and clear differences between the pollen season and the subsequent pollen-free period (out of season) in Figure 3.

**Figure 4:**
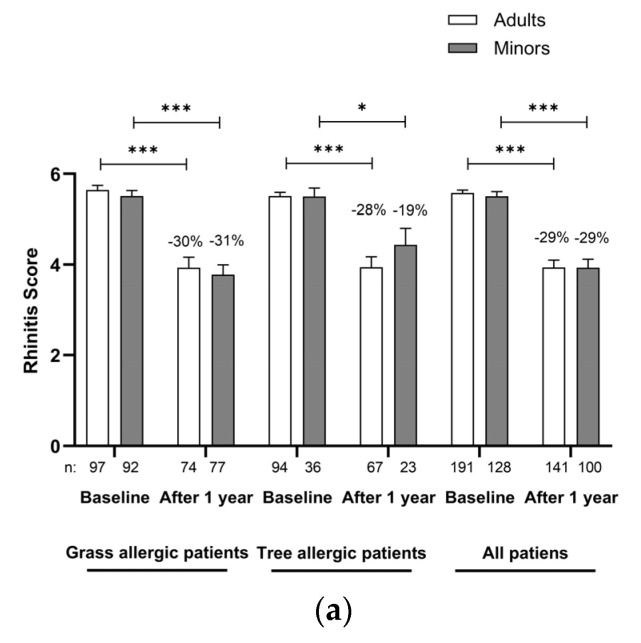

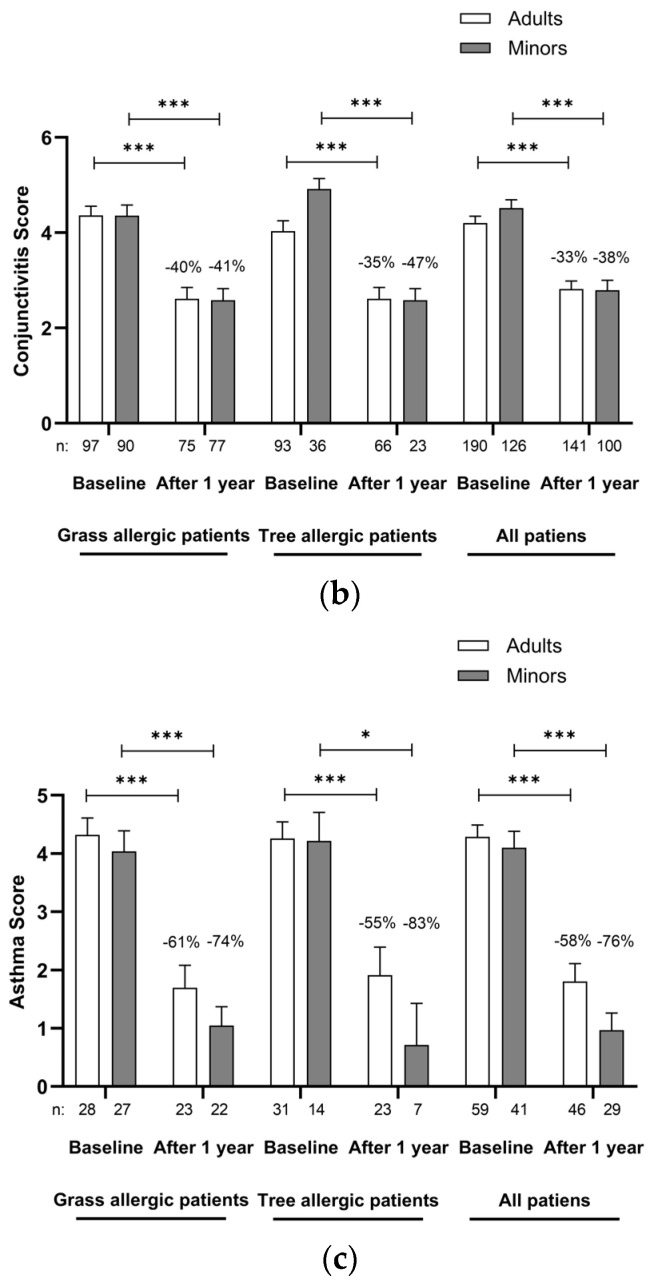
(**a**). Rhinitis Score at baseline and after 1 year of treatment in the adults and minors group. Data are expressed as mean and SEM. * *p* < 0.05, *** *p* < 0.001 in comparison to baseline. SEM, standard error of mean. (**b**)**.** Conjunctivitis Score at baseline and after 1 year of treatment in the adults and minors group. Data are expressed as mean and SEM. *** *p* < 0.001 in comparison to baseline. SEM, standard error of mean. (**c**). Asthma Score at baseline and after 1 year of treatment in the adults and minors group. Only asthmatic patients were considered for the Asthma Score. Data are expressed as mean and SEM. * *p* < 0.05, *** *p* < 0.001 in comparison to baseline. SEM, standard error of mean.

In the retrospective survey of rhinitis, conjunctivitis and asthma symptoms according to Sieber’s symptom score, the effects of the therapy are already evident in the first year in all patients and in both homologous allergen groups (Figure 4a–c).

The mean rhinitis score after one year of AIT was significantly reduced compared to baseline by 29% in all patients (*p* < 0.001) with no significant difference between the age groups. The improvement was similar in both homologous allergen groups. The results for the conjunctivitis were 33% in adults versus 38% in minors, again with no significant differences either for age or for the homologous allergen groups. The asthma score significantly (*p* < 0.001) improved in asthmatic minors by 76% compared to 58% in adults. Again, the improvement was comparable in grass-allergic and tree-allergic patients after one year of AIT.

### Safety and Tolerability

In this NIS, a total of 653 adverse events were documented in the first year. Of these, 381 were classified by the investigators as related to the therapy (adverse drug reactions, ADR). Data of patients with ADRs are shown in Table 3.

These ADRs affected 29 out of 190 patients with grass pollen allergy and 22 out of 130 patients with tree pollen allergy. In total, this corresponds to around 15% of the population, which conversely means that 85% of the patients treated did not develop any side effects during therapy. Slightly more minors compared to adults in both homologous allergy groups were affected by side effects. In both homologous groups, the side effects were predominantly of a local nature, as is usual with the subcutaneous application of therapeutic allergens. It is a common finding that reporting culture in minors is higher compared to adults.

In 22 patients, 109 systemic side effects were documented, with equal incidence in adults and minors. There were no fatalities, nor was adrenaline used to control these systemic side effects in any patient. However, one of these systemic reactions in adults was classified as serious, but the patient recovered quickly under medical treatment without need for hospitalisation overnight (narrative provided as Supplementary file S1). To summarise, one in 15 injections of these allergoids resulted in a local reaction, while one in 80 injections was associated with a systemic reaction. The latter occurred in about 0.8% of all patients.

## 4. Discussion

The frequency of allergies in children [26] and high sensitisation rates as well as the increasing trend [27] underline the importance of paediatric studies. The EAACI AIT guideline [2] calls for prospective studies in children and explicitly also long-term data in children. Alvaro-Lozano et al. (2020) [28] pointed out that more studies to determine the effectiveness and long-term benefit are lacking. They state that more clinical trials are required to confirm and validate the efficacy and long-term clinical benefits of AIT in children. Moreover, there are fewer paediatric data compared to adults. Caffarelli et al. (2020) [29] explain that there are few data on long-term efficacy in children, and the existing studies only have small numbers of patients.

A further rationale for the justification of this cohort study is that few prospective data exist for the perennial use of MATAs, besides the published RWE data [30]. Therefore, it is the aim of this non-interventional study to investigate the long-term effects of an MATA treatment in patients suffering from allergic rhinoconjunctivitis with or without asthma caused by grass pollen or tree pollen. According to general recommendations in allergy and the summary of product characteristics (SmPCs), patients are following a three-year SCIT treatment course, followed by a two-year observation period. Patients record their allergy symptoms and the consumption of anti-allergic symptomatic medication using an electronic diary at the peak of their respective pollen seasons over the full course of the five-year study. This study was designed as a cohort study of adults versus minors in order to investigate the effectiveness of the MATAs in children in more depth. The design of this study is presented here, and the results of the first year of treatment are described.

To our best knowledge, this study is the largest long-term study on pollen allergy in which paediatric patients are explicitly compared with adults regarding the efficacy, safety and tolerability of subcutaneous allergen-specific immunotherapy. One of the strengths of this study is that a sufficient number of patients from both age groups were examined, although a slight disbalance between the groups remained, to allow generalised and robust conclusions to be drawn about the outcome of the therapy. This is partly due to the fact that the preparations used are products that have been authorised in Germany for decades and are indicated for children from the age of five. There are, however, also limitations in this approach: the observational cohort design, comparing paediatric patients to an adult comparator group, inherently introduces potential biases. Baseline differences between children and adults, such as disease severity, treatment history, or other confounding factors, may have impacted results. This non-randomised comparison could be influenced by unmeasured confounders, limiting the validity of the conclusions. These current findings represent preliminary data, limiting conclusions regarding long-term efficacy or potential disease-modifying effects, and conclusions regarding long-term outcomes must be deferred pending further data.

Unlike in other long-term studies [8,31,32], or the large paediatric double-blind GCP studies on grass pollen allergy [33,34] no sublingual tablets were used here. Although these allow the use of placebos in a simple manner, blinding is rather questionable due to the local side effects in the oral cavity that occur in almost all actively treated patients [35,36]. Furthermore, a GCP setting of a DBPC clinical trial always represents a somewhat artificial setting, whereas NIS studies are closer to a real-world situation. The long-term PAT study [3] is therefore most suitable for comparison, although fewer than 100 children were treated with subcutaneous therapy over three years. This study also deliberately avoided placebo and blinding but was randomised, including a control group that was treated exclusively with symptomatic therapy. Eng’s long-term study on little more than 10 actively treated patients was designed in a similar way, in which the control group also received symptomatic therapy [37,38]. In contrast to this, in our cohort study, an adult population was used as the control group, which therefore received an effective evidence-based AIT therapy. Whatever the case, characteristics of a high evidence level, such as randomisation, use of placebos and/or blinding, can be seen as a design weakness of our approach. In other areas of medicine, however, such cohort studies have long been quite common and are regarded as meaningful by being closer to the real-life setting [39]. In a recently published systematic review the authors point out the lack of well-designed studies to support the evidence for primary and secondary prevention of AIT in respiratory allergy [40].

The results of all effectiveness parameters of the first year of treatment underpin the essential hypothesis of this study, namely the non-inferiority of treatment success in children/adolescents compared to adults. In particular, the primary target parameter shows that the daily recorded score of a combination of allergic symptoms and medication consumption in children with high allergen exposure in the season is even marginally more favourable compared to adults. This is in line with the results of two large double-blind paediatric trials using sublingual tablets [33,34]. In grass pollen-allergic patients, the contribution of the symptoms to the CSMS is almost twice as large as that of the medication. This imbalance is even more pronounced in tree pollen-allergic patients. Here, the contribution of the symptoms is almost three times as large as that of the medication. In children, this disparity in symptoms and medication consumption is explainable by caregivers’ intervention and has already been demonstrated in double-blind placebo-controlled randomised prospective studies [28].

The improvement in symptom burden in the retrospective assessment by patients is particularly impressive and in line with previously published data [4], but this parameter is discussed controversially and is susceptible to recall bias. With regard to disease-related quality of life, it can generally be stated that the restrictions due to allergic symptoms are relatively low even after the first treatment cycle, although the contrast is less pronounced in children than in adults. This may be partly due to the lower sensitivity of an external assessment by the caregivers [15]. The careful documentation of adverse events and, in particular, of local and systemic side effects carried out in this study does not lead to any new findings. It is similar to the findings of the meta-analysis by Becker [11]. The higher incidence of adverse drug reactions in minors can be explained by the special attention of caregivers in the context of a clinical trial. This was reported before [41]. With 85% of patients completing the first therapy cycle without any ADR, the MATA platform used here can be described as safe to use and well tolerated, especially when compared with the frequent local side effects of sublingual therapy [8].

The guideline on allergy immunotherapy for IgE-mediated allergic diseases [9] points out that the efficacy of SCIT with grass pollen extracts in seasonal allergic rhinoconjunctivitis and seasonal allergic asthma in children has only been proven by a few studies. For tree pollen allergy, however, there are no specific studies in children and adolescents that could prove the efficacy of SCIT. There is only limited evidence from a few (combined) studies for the efficacy and safety of the therapy at this age. Therefore, the clinical implications of our cohort study, which includes both grass and tree pollen allergy sufferers in children and adolescents as well as adults, could be to bridge major knowledge gaps existing today. If the hypothesis of an equivalence of effect in both age groups proves to be true, not only in the first year of therapy but also in the long term after discontinuation of treatment, this could lead to greater acceptance of these only causal forms of treatment for IgE-mediated allergy. Despite all the limitations of this study, there is therefore an opportunity to provide clear evidence of the efficacy of subcutaneous AIT in pollen allergy by demonstrating continued efficacy even in the second year after treatment, which has not yet been available in this form and, in particular, in the size of the observed cohort. This would provide strong arguments in favour of allowing more minors to benefit from this time-consuming and resource-intensive treatment method.

## 5. Conclusions

In summary, despite its limitations, like potential baseline biases or unmeasured confounders limiting the validity of the conclusions, this study approach of a cohort study has yielded promising results by this first interim analysis for the initial course of therapy in the first year. However, the basic hypothesis of non-inferiority of the long-term therapeutic effects of the MATA platform will be addressed at the end of the two-year follow-up period. This will be the subject of a further publication.

## Figures and Tables

**Table 1:** Baseline characteristics.

Age Group		Minors		Adults		Total	
		Count	%	Count	%	Count	%
Allergic diseases							
Allergic rhinitis Severity	Mild	9	7.1%	9	4.7%	18	5.7%
	Moderate	54	42.5%	88	46.1%	142	44.7%
	Severe	64	50.4%	94	49.2%	158	49.7%
	Total	127	100.0%	191	100.0%	318	100.0%
Allergic conjunctivitis							
Severity	Mild	19	16.7%	43	25.9%	62	22.1%
	Moderate	50	43.9%	78	47.0%	128	45.7%
	Severe	45	39.5%	45	27.1%	90	32.1%
	Total	114	100.0%	166	100.0%	280	100.0%
Allergic asthma							
Severity	Mild	18	42.9%	31	49.2%	49	46.7%
	Moderate	16	38.1%	20	31.7%	36	34.3%
	Severe	8	19.0%	12	19.0%	20	19.0%
	Total	42	100.0%	63	100.0%	105	100.0%
Sensitisation Profiles							
Monosensitised		51	39.8%	74	38.9%	125	39.3%
Polysensitised		77	60.2%	116	61.1%	193	60.7%
Total		128	100.0%	190	100.0%	318	100.0%
Main allergen + grasses		11	14.3%	33	28.4%	44	22.8%
Main allergen + trees		26	33.8%	15	12.9%	41	21.2%
Main allergen + animal epithelia		26	33.8%	33	28.4%	59	30.6%
Main allergen + house dust mite		39	50.6%	54	46.6%	93	48.2%
Main allergen + mould fungi		7	9.1%	16	13.8%	23	11.9%
Main allergen + other		13	16.9%	36	31.0%	49	25.4%
Concomitant medications							
Cases		114	88.4%	153	80.1%	267	83.4%
Eye drops		72	55.8%	64	33.5%	136	42.5%
Antihistamines: oral		95	73.6%	128	67.0%	223	69.7%
Corticosteroids: oral		5	3.9%	6	3.1%	11	3.4%
Corticosteroids: nasal		47	36.4%	49	25.7%	96	30.0%
Antihistamines: nasal		38	29.5%	30	15.7%	68	21.3%
Corticosteroids: inhaled		20	15.5%	24	12.6%	44	13.8%
ß-sympathomimetics: inhaled		26	20.2%	17	8.9%	43	13.4%
Other		6	4.7%	18	9.4%	24	7.5%
Concomitant diseases							
Cases		48	37.2%	66	34.6%	114	35.6%
Atopic dermatitis		28	21.7%	11	5.8%	39	12.2%
Food allergy/intolerance		20	15.5%	25	13.1%	45	14.1%
Urticaria		5	3.9%	0	0.0%	5	1.6%
Nasal polyposis		3	2.3%	2	1.0%	5	1.6%
ASA intolerance		0	0.0%	1	0.5%	1	0.3%

**Table 2:** Age distribution of the main allergen (the target of AIT in this study) of the participants.

Age Group	Main Allergen	Frequency	Percentage
Children (5–11 years)	Grasses	56	71.8
	Trees	22	28.2
	Total	78	100
Adolescents (12–17 years)	Grasses	37	72.5
	Trees	14	27.5
	Total	51	100
Adults (18–75 years)	Grasses	97	50.8
	Trees	94	49.2
	Total	191	100
Total	Grasses	190	59.4
	Trees	130	40.6
	Total	320	100

**Table 3:** Adverse drug reactions during the first year of the treatment. (ADR, adverse drug reaction; * Chi-square test).

Total Patients with at Least One ADR (n = 320)	Minors (n = 129)	Adults (n = 191)	*p*-Value
None	101 (78.3%)	168 (88.0%)	0.021 *
Not serious	28 (21.7%)	22 (11.5%)	
Serious	0 (0.0%)	1 (0.5%)	
Local	26 (20.2%)	16 (8.4%)	0.002 *
Systemic	10 (7.8%)	14 (7.3%)	0.888 *

## Data Availability

Upon request, data will be available from the corresponding author.

## Supplementary Materials

The following supporting information can be downloaded at: https://www.mdpi.com/article/10.3390/jcm14082609/s1, Table S1: Overview of the design of this study with the main assessment parameters recorded and the times at which they were collected. Table S2: Dropouts first year. Table S3: Reasons for withdrawal from this study. S1: Narrative SAE. S2: Product information TA top Gräser/Bäume.

## Abbreviations

The following abbreviations are used in this manuscript:

ACT	Asthma Control Test
ADR	Adverse Drug Reaction
AE	Adverse Event
AIT	Allergen-specific immunotherapy
AR	Allergic Rhinitis
CSMS	Combined Symptom and Medication Score
DBPC	Double-blind, placebo-controlled
dMS	Daily Medication Score
dSS	Daily Symptom Score
DWD	German weather service
EAACI	European Academy of Allergy and Clinical Immunology
EMA	European Medicines Agency
GCP	Good Clinical Practice
HTTPS	Hypertext Transfer Protocol Secure
MATA	Microcrystalline Tyrosine-associated Allergoid
MCT	Microcrystalline Tyrosine
NIS	Non-Interventional Study
PIP	Paediatric Investigation Plan
RCT	Randomised Controlled Trial
RCAT	Rhinitis Control Test
RQLQ	Rhinoconjunctivitis Quality of Life Questionnaire
SAE	Serious Adverse Event
SCIT	Subcutaneous allergen-specific immunotherapy
SLIT	Sublingual allergen-specific immunotherapy
SmPCs	Summary of Product Characteristics
VAS	Visual Analogue Scale
