# Correction: Gerstlauer et al. TAPAS—A Prospective, Multicentre, Long-Term Cohort Study in Children, Adolescents and Adults with Seasonal Allergic Rhinitis—Design and Early Results. *J. Clin. Med.* 2025, *14*, 2609

**DOI:** 10.3390/jcm14248727

**Published:** 2025-12-10

**Authors:** Michael Gerstlauer, Julia Hiller, Jennifer Raab, Katrin Birkholz, Martin Tapparo, Christian Neuhof, Laura Day, Anna Rybachuk, Cengizhan Acikel, Hacer Sahin, Kim Hebbeler, Sven Becker, Christian Vogelberg, Silke Allekotte, Matthias F. Kramer, the TAPAS Study Group

**Affiliations:** 1 Paediatric and Adolescent Medicine, Department of Paediatric Pneumology and Allergology, University Medical Center Augsburg, 86156 Augsburg, Germany; 2 Bencard Allergie GmbH, 80804 Munich, Germany; 3 ClinCompetence Cologne GmbH, 50668 Cologne, Germany; 4 Institute of Medical Statistics and Computational Biology, University of Cologne, 50923 Cologne, Germany; 5 Department of Otorhinolaryngology, Head and Neck Surgery, University of Tübingen, 72074 Tübingen, Germany; 6 Department of Pediatric Pneumology and Allergology, Faculty of Medicine, University Hospital Carl Gustav Carus Dresden, Technische Universität Dresden, 01069 Dresden, Germany; 7 Allergy Therapeutics, Worthing BN14 8SA, UK

## Error in Figure

In the original publication [1], there was a mistake in Figure 3 as originally published. The authors have identified mistakes in the calculation of the parameter RQLQ for minors and adults and have therefore corrected Figure 3, in which these data appear. The corrected Figure 3 appears below. The legend to the Figure remains the same.

The authors state that the scientific conclusions are unaffected. This correction was approved by the Academic Editor. The original publication has also been updated.

## Figures and Tables

**Figure 3:**
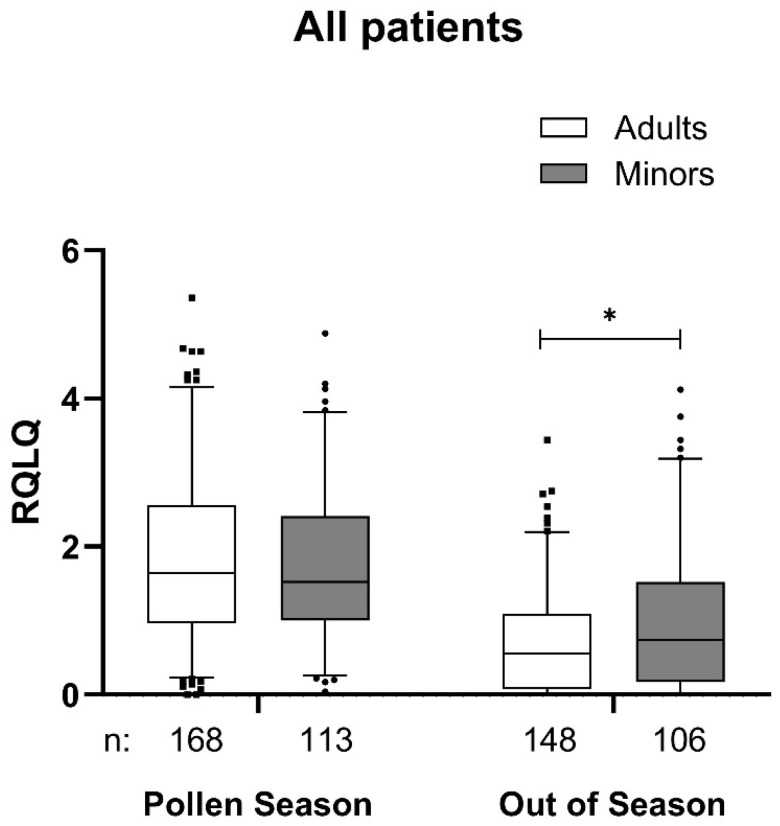
RQLQ Score at the first pollen season in 2021 or 2022 and out of season in the adults and minors group. Data are presented as a box-whisker plot with 5 and 95 percentiles. * *p* < 0.05 in comparison to adults. RQLQ, rhinoconjunctivitis quality of life questionnaire.
