# Supplementary material for: TAPAS—A Prospective, Multicentre, Long-Term Cohort Study in Children, Adolescents and Adults with Seasonal Allergic Rhinitis—Design and Early Results
Source: J Clin Med. 2025 Apr 10;14(8):2609. doi: 10.3390/jcm14082609 (PMC12027696; doi:10.3390/jcm14082609)
Supplement: Supplementary file 1 [file jcm-14-02609-s001.zip › jcm-3565040-supplementary.pdf]

Journal of
Clinical Medicine

MDPI

Table S1. Overview of the design of the study with the main assessment parameters recorded and the times at which they were collected.

	Inclusion visit	Treatment phase Year 1 - 3			Observation phase Year 4 + 5	
		**Regular injection visits**	**Visit at the peak or directly after the peak of the pollen season[1]**	**Visit in autumn[2]**	**Visit at the peak or directly after the peak of the pollen season[1]**	**Visit in autumn[2]**
Declaration of consent to the collection and use of data	■					
Demographic data; body measurements	■					
Allergological anamnesis	■					
Diagnostic tests (SPT, IgE, provocation tests)[3]	■					
Documentation of SCIT with TA Grasses top or TA Trees top (dose, dose adjustments)		■	■	■		
Documentation side effects of SCIT		■	■	■		
Documentation by the doctor						
Retrospective documentation of the severity of allergic symptoms and use of anti-allergic medication during the previous pollen season	■			■		■
Documentation by the patient						
Daily documentation of symptom severity and intake of anti-allergic medication using the CSMS+ Diary App			■[4]		■	
Quality of life documentation (RQLQ)			■	■	■	■
Documentation of rhinitis control (RCAT)			■	■	■	■
Documentation of asthma control (C-ACT/ ACT)[5]			■	■	■	■
Documentation of side effects		■	■	■		
Assessment of tolerance by the doctor				■[6]		
Assessment of satisfaction with the therapy (doctor)				■[6]		■

[1] For patients treated with TA Trees: April, for patients treated with TA Grasses: June

[2] Preferably in November/December (preparation-independent)

[3] Only those diagnostic tests that are routinely used in standard care are documented.

[4] Documentation over the entire pollen season

[5] Documentation only if asthma is present.

[6] The doctor's assessment during the treatment phase is documented once after 3 years of treatment.

Table S2. Dropouts first year.

Age set		Frequency	Percent	Valid Percent	Cumulative Percent
**Minors**	No	115	89.1	89.1	89.1
	Yes	14	10.9	10.9	100
	Total	129	100	100	
**Adults**	No	160	83.8	83.8	83.8
	Yes	31	16.2	16.2	100
	Total	191	100	100	
**Total**	No	275	85.9	85.9	85.9
	Yes	45	14.1	14.1	100
	Total	320	100	100	

Table S3. Reasons for withdrawal from the study.

Dropouts 1st year (N=45)			
**Reasons**	**Numbers**		
	Total	Adults	Minors
Other non-drug-related reasons	21	18	3
Too much time required for the therapy	11	6	5
Relocation of the patient	6	4	2
Occurrence of non-tolerable side effects	4	3	1
Occurrence of a contraindication (e.g. pregnancy)	2	2	0
Lost to follow-up	1	1	0

S1. Narrative SAE.

A 27-year-old female participating in the non-interventional TAPAS (Tyrosine Allergoid Paediatric and Adult Study) study received TA GRÄSER TOP GRUND- UND FORTSETZUNGS BEHANDLUNG (Grasses/Rye 100%) 2000 SU, 0.5 mL vial 3. Before this injection, a short infusion of vitamin C was also administered at the general practitioner in the morning to relieve recurrent herpes virus outbreaks.

After the injection, the patient experienced severe sweating, feeling hot, severe facial redness, severe pressure on her chest, itching of hands, severe defecation urgency and burning eyes. Anaphylactic reaction was reported and considered as medically important and serious. The patient was treated with 200 mg Prednisolone infusion and Dimethindenemaleat (antihistamine). The patient was transported to the emergency unit where she was reported as fully recovered. Neither dyspnoea no stridor was observed. Vital signs (heart rate, RR, body temperature and SpO2) were normal. The patient was discharged on the same day. Of note, overnight hospitalization was not indicated. This case demonstrates that augmentation factors must be taken into account for each injection as infections or vitamin C can play a role in the induction of anaphylactic reactions during AIT. Causality was classified as probable, but is never clear when two drugs are administered. The patient was fully recovered and continued the study.

S2. Product information TA top Gräser/Bäume

(Extract from Summary of Product Characteristics February 2017)

Initial and continuation course: 600, 1600, 4000 Standardised Units (SU)/ml; Suspension for injection

Vial/pre-filled syringe No./label colour	Dose (SU/ml)
1 Green	600
2 Yellow	1600
3 Red	4000

Qualitative and Quantitative Composition TA top Bäume:

TA Trees top contains purified allergen extracts which were chemically modified and L-tyrosine-adsorbed of the following pollens (early flowering trees) in equal parts:

Birch (Betula spp.)

Alder (Alnus spp.)

Hazel (Corylus spp.)

Qualitative and Quantitative Composition TA top Gräser:

TA Grasses top contains purified allergen extracts which were chemically modified and L-tyrosine-adsorbed of the following pollens (grass and rye) in equal parts:

Rye (Secale cereale)

Meadow foxtail (Alopecurus pratensis)

Dogstail (Cynosurus cristatus)

Cocksfoot (Dactylis glomerata)

Perennial rye (Lolium perenne/multiflorum)

Smooth grass (Poa pratensis/trivialis)

Sweet vernal grass (Anthoxanthum odoratum)

Meadow fescue (Festuca pratensis)

Common bent grass (Agrostis tenuis/capillaris)

Timothy grass (Phleum pratense)

Brome (Bromus spp.)

False oat grass (Arrhenatherum elatius)

Yorkshire fog (Holcus lanatus)

Treatment regimen:

Initial course

A total of 3 injections, one from each of the 3 vials/pre-filled syringes contained in the initial course (No. 1 (green) to No. 3 (red)), should be administered. The table below illustrates the recommended treatment regimen.

Vial / pre-filled syringe No. and colour	Recommended posology	Alternative posology*	Interval from previous injection (weeks)	
			Min	Max
No. 1 (Green)	300 SU – 0.5 ml	120 SU – 0.2 ml	1	2
		300 SU – 0.5 ml		
No. 2 (Yellow)	800 SU – 0.5 ml	320 SU – 0.2 ml	1	2
		800 SU – 0.5 ml		
No. 3 (Red)	2000 SU – 0.5 ml	800 SU – 0.2 ml	1	2
		2000 SU – 0.5 ml		

* Alternative posology e.g. for highly sensitised patients (only for vials)

Continuation course

After the initial course, the treatment should be continued in order to ensure and increase the success of therapy. At least 3 injections each with 0.5 ml of vial/pre-filled syringe No. 3 (2000 SU) should be administered.

Vial / pre-filled syringe No. and colour	Recommended posology	Interval from previous injection (weeks)	
		Min	Max
No. 3FF (Red) / No. 3 (Red)	2000 SU – 0.5 ml	1	4 (6)
	2000 SU – 0.5 ml	1	4 (6)
	2000 SU – 0.5 ml	1	4 (6)
